# A New Method for Detecting Spurious Musk-Pods

**Published:** 1846-06

**Authors:** J. Moore Neligan

**Affiliations:** Physician to Jervis street Hospital, &c.


*A New Method for detecting Spurious Musk-Pods.* By J. Moore Neligan, M. D., M. R. I. A., Physician to Jervis street Hospital, &c.—Owing to the high price and great demand for musk, both as a medicine and a perfume, it is very generally much adulterated. This fact is so well known to apothecaries and to druggists, that those who have even a moderate consumption of the drug, prefer purchasing it in the unopened musk-bag, or, as it is technically called, *musk-pod.* This precaution, however, is often found not to be a sufficient protection against fraud, as spurious musk-pods are not uncommon in commerce, so well prepared that even the most experienced eye is often unable to distinguish the true from the false.

It is now very generally known, that musk is the peculiar secretion of a small sac situated immediately in front of the preputial orifice of the male musk animal, the *Moschus moschiferus,* and that it is principally imported into the British market from China. The Chinese, finding a greater demand for musk than they are able to supply with the genuine article, squeeze out some of the secretion, which is fluid in the recent state, and mix it with, it is believed, the dried blood of the animal; this compound, which presents the same physical characters as true musk, they put into small sacs made of pieces of the skin cut off from other parts of the animal's body and prepared with the usual ingenuity of this people, so much so, indeed, as almost to defy detection with the naked eye.

The method hitherto adopted for detecting this sophistication, has been the peculiar position of the hairs, which are arranged in a circular manner around the orifice in the genuine musk-pod, and also the absence of any remains of the penis in the artificial pods. But those characters are not invariable, and I have seen some spurious musk-pods which were so skilfully prepared as to be undistinguishable from the genuine article when compared with them.

The plan which I propose, depends on the microscopic characters of the hairs which grow on the preputial sac of the musk animal, and which, as far as I have been able to detect by direct experiment, differ very remarkably from those of the false sacs which are met with in commerce. This test I have recently had an opportunity of pointing out to my friend Professor Christison, of Edinburgh, and of illustrating it to him from specimens in his own museum.

The difference appears to depend on the fact, that the hairs of this part of the animal are furnished in the interior with distinct, regular, colour cells, while in hairs taken from other parts of the animal's body those cells appear to be obliterated, as is generally the case in this and the allied tribes of animals.

The method I have now proposed is a very simple one, and of easy application, and cannot be considered too scientific in the present day, when every pharmaceutist must be supposed to be provided with a microscope at the least of the power above spoken of, without which he could not possibly detect the adulteration of arrow root and of the other feculas of commerce.—*Dublin Journ. of Med. Science.*

